# Smart integrated biomaterial systems for precision and optimized delivery of MSCs and their exosomes: Transforming wound healing and organ regeneration

**DOI:** 10.1016/j.reth.2025.101045

**Published:** 2025-11-26

**Authors:** Fatemeh Rafati, Zeynab Ghorbani, Tahereh Manoochehrabadi, Farbod Khosravi, Jila Majidi, Fatemeh Eskandari, Mohamad Eftekhary, Hajar Nasiri

**Affiliations:** a Department of Materials and Metallurgical Engineering, Amirkabir University of Technology (Tehran Polytechnic), Tehran, Iran; b Medical Biotechnology Research Center, School of Paramedicine, Guilan University of Medical Sciences, Rasht, Iran; c Department of Anatomical Sciences, Faculty of Medicine, Guilan University of Medical Sciences, Rasht, Iran; d Tissue Engineering Research Group, Department of Anatomy and Cell Biology, School of Medicine, Mashhad University of Medical Sciences, Mashhad, Iran; e School of Medicine, Shahid Beheshti University of Medical Sciences, Tehran, Iran; f Department of Tissue Engineering & Regenerative Medicine, Faculty of Advanced Technologies in Medicine, Iran University of Medical Sciences, Tehran, Iran; g Department of Tissue Engineering and Applied Cell Sciences, School of Paramedicine, Guilan University of Medical Sciences, Rasht, Iran

**Keywords:** Mesenchymal stem cells, Exosomes, Biomaterial, Nanoparticle, Hydrogels, Smart wound dressings

## Abstract

Mesenchymal stem cells (MSCs) and their exosome (MSCs-Exos) have been shown to be major therapeutic candidates in regenerative medicine due to their inherent capacity for modulating immune response, promoting angiogenesis, and repairing tissues. However, clinical application of MSCs and MSCs-Exos is hindered by various intrinsic barriers, such as poor survival of transplanted MSCs, quick systemic clearance of exosomes, uncontrolled release of therapeutic payloads, and loss of function in severe pathological situations, such as chronic wounds and fibrotic tissues. To overcome these limitations, recent advances have focused on the design of modern delivery systems to enhance the stability, bioavailability, and functionality of MSCs and MSCs-Exos. These platforms include responsive hydrogels, engineered nanoparticles, and multi responsive intelligent dressings that mirror protective microenvironments in general and provide controlled, long-term distribution of bioactive elements. This current review focuses on such innovative approaches that enhance regeneration results in strong preclinical models, maximize therapeutic delivery, and boost MSCs and exosome survival. Despite remarkable advancements, major obstacles still exist, such as guaranteeing biosafety, achieving scale production, and obtaining regulatory approvals. The combination of MSCs and MSCs-Exos therapies with intelligent, responsive biomaterials capable of providing antimicrobial function and active monitoring has the potential to revolutionize tissue regeneration and wound healing and make MSCs and MSCs-Exos invaluable resources in precision regenerative medicine of the future.

## Graphical abstract

**Figure:**
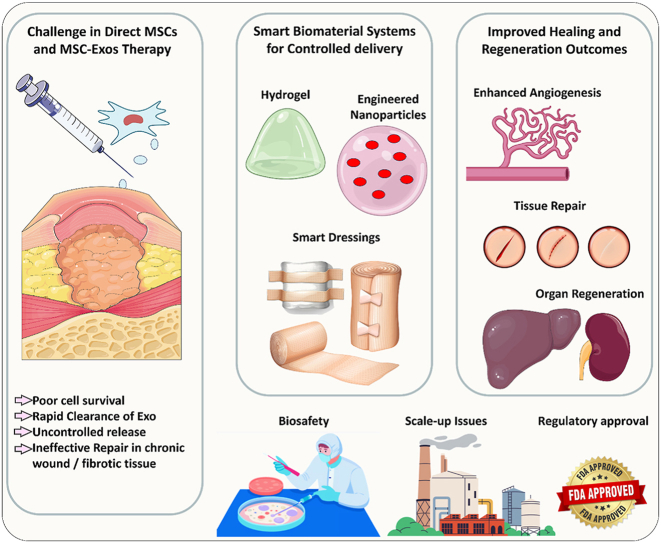

## 1 Introduction

Mesenchymal stem cells (MSCs) and their secreted exosomes (MSCs-Exos) have emerged as powerful tools in regenerative medicine with remarkable functions in diseases ranging from pulmonary fibrosis and myocardial infarction to chronic wounds. The therapeutic potential of MSCs and MSCs-Exos is mainly related to their unique and powerful abilities to regulate immune responses, promote angiogenesis, and also orchestrate tissue regeneration. However**,** clinical translation of MSCs and MSCs-Exos based therapies remains hampered by some challenges such as survival limitations, rapid MSCs-Exos clearance, and regulation of therapeutic release in pathological environments. To alleviate these limitations, advanced biomaterial systems, including nanoparticles, stimulus-responsive hydrogels, and smart wound dressings, have been designed to sustain and protect MSCs and MSCs-Exos by allowing controlled and targeted delivery [1]. These biomaterials increase the effects of regeneration processes by improving stability and bioavailability, as well as by more closely resembling the extracellular matrix. Integrating these technologies to optimize the delivery of MSCs and MSCs-Exos is a significant step toward precision regenerative medicine. Recent developments have brought attention to the potential of MSCs and their exosomes as prospective adjuvants in transplantation such as hematopoietic stem cell transplantation (HSCT) settings as well as in regenerative medicine [2]. Their anti-inflammatory and immunomodulatory properties can improve tissue repair after transplantation, lower post-transplant problems, and increase graft acceptability [3,4]. Therefore, combining MSC-derived exosomes with intelligent biomaterial-based delivery systems may offer innovative therapeutic approaches that connect transplant immunotherapy and regenerative healing. This review emphasizes current advances in biomaterial-assisted MSCs and exosome therapies, with a particular focus on their application in wound healing and the creation of next-generation smart dressings.

## 2 Mesenchymal Stem Cells and MSCs-derived exosomes (MSCs-Exos)

### 2.1 Main characteristics and capabilities

MSCs are multipotent cells that can differentiate into many cell lineages and have anti-inflammatory, antifibrotic, antibacterial, and immunomodulatory properties [5,6]. MSCs are a subset of fibroblast-like pluripotent cells that, regardless of injection route technique, can move toward injured regions [7,8]. MSCs have the potential to be a novel treatment for a number of illnesses due to their multipotent traits, migratory ability, and immune-privileged status. The International Society for Cellular Therapy (ISCT) has set basic standards for MSCs from different sources, including adhesion to plastic, expression of CD105, CD73, and CD90, absence of hematopoietic markers CD34 and CD45, and the ability to differentiate into three lineages under normal circumstances. Both adult and embryonic sources can produce human MSCs, while embryonic MSCs have a higher capacity for differentiation and flexibility [9]. Although bone marrow is the main source that is advised for the extraction of these cells, they can also be obtained from other sources such as newborn blood, umbilical cord blood, wharton's jelly, adipose tissue, amniotic fluid, olfactory mucosa, and endometrial stem cells generated from menstrual blood [10,11]. Despite biological heterogeneity linked to their origin, MSCs generally indicate similarities in immunophenotypes, differentiation potential, and morphology [12]. The non-immunogenicity of MSCs is a fundamental characteristic, as evidenced by the expression of MHC class I and the absence of MHC class II, which induces the inactivation of T cells and the development of immunosuppressive effects. This finding facilitates allogeneic transplantation of MSCs without the requirement for immunosuppression and rejection [13].

Furthermore, MSCs demonstrate minimal immunogenicity and considerable immunomodulatory potentials, in terms of the lack of proliferative response from allogeneic lymphocytes [14]. Their immunomodulatory capabilities make them promising therapeutic candidates for treating tissue damage and inflammation [15]. Oxidative stress and chronic inflammation are known as key factors for organ fibrosis [16]. However, due to decreased abilities of MSCs by *in vitro* proliferation or after transplantation, it is critical to coordinate formulating strategies to improve the clinical performance of MSCs. The challenges, such as inadequate migration to injury sites, poor homing, difficulties in differentiation during prolonged culture, and adverse microenvironmental conditions, highlight the necessity for comprehensive MSCs optimization [11].

**Fig. 1:**
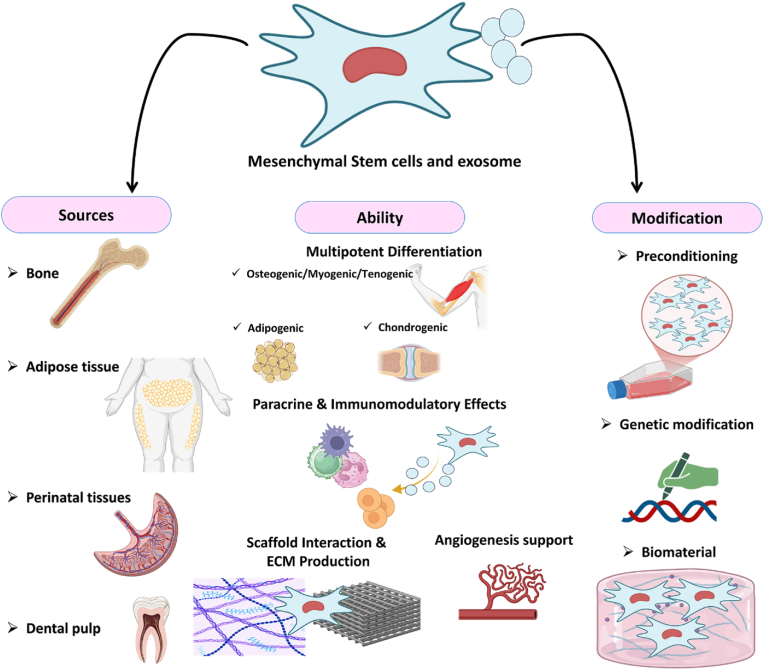
**Schematic overview of MSCs and MSCs-Exos.** Different sources of MSCs and MSCs-Exos display various abilities; however, optimization by a proper strategy of modifications enhances their naïve potentials. The figures are original and were created using Microsoft Office PowerPoint 2021.

MSCs-derived exosomes (MSCs-Exos) are nano-sized extracellular vesicles that contain proteins, lipids, and nucleic acids, and can regulate the immune system. All cell types release extracellular vesicles (EVs) into the environment, including exosomes, microvesicles, and apoptotic bodies, all of which are enclosed by a lipid bilayer. The classification of EVs is mainly size-based: exosomes (30–150 nm), microvesicles (200–1000 nm), and apoptotic bodies (800–5000 nm) [17]. MSCs-Exos can cross biological barriers and deliver their molecular cargos directly to target cells, thereby influencing cell survival, growth, and function. When applied at the injury site, MSCs-Exos often achieve more favorable outcomes than direct MSCs therapy by enhancing paracrine signaling and promoting differentiation [18]. MSCs-Exos offer several therapeutic advantages, including precise modulation of immune responses, promotion of angiogenesis, and enhancement of tissue repair mechanisms. Their nanoscale size facilitates targeted delivery, prolonged circulation, and efficient cellular uptake. Additionally, exosomes can be engineered or preconditioned to carry bioactive molecules, further amplifying their regenerative potential [19]. Similar to their parental MSCs, EVs are characterized by low immunogenicity, circulatory stability, biocompatibility, and the ability to traverse biological barriers [20]. Therapies using MSCs and MSCs-Exos have demonstrated significant promise in treating a wide range of conditions, including pulmonary fibrosis, chronic wounds, and neurodegenerative disorders. However, MSCs-based therapies are still hindered by challenges such as low cell viability, poor engraftment, rapid clearance, and immune-mediated rejection *in vivo*. Likewise, the clinical translation of MSCs-Exos is limited by several unresolved issues, including the lack of standardized purification methods, heterogeneity of EVs subtypes with diverse molecular cargos, and uncertainties surrounding the most effective administration routes, dosage regimens, therapeutic efficacy, and cost-effectiveness [21]. Fig. 1 illustrates sources, abilities, and available strategies for optimization of MSCs and MSCs-Exos.

Although MSCs share a common immunophenotype and multipotent differentiation potential, growing evidence highlights their heterogeneity in biological behavior and therapeutic potency. Such variability arises mainly from three interconnected factors: tissue source, donor characteristics, and culture condition [22]. MSCs from different sources (e.g., bone marrow, adipose tissue, umbilical cord, wharton's jelly, placenta) show clear differences in the transcriptomic and secretory profile, shaping their immunomodulatory capacity and the signature of their released exosomes. For example, MSCs derived from adipose tissue frequently present higher proliferative rates and distinct cytokine or miRNA expression profiles compared with bone-marrow-derived MSCs, leading to differences in the regenerative outcome [23,24]. Likewise, donor parameters, such as age, sex, metabolic status, and genetic background, may influence the proliferation of MSCs, their cytokine production, and miRNA content of exosomes, leading to therapeutic variability [25]. Moreover, *in* *vitro* parameters, such as oxygen tension, passage number, media composition, and preconditioning with physical/chemical/biologic agents, strongly impact MSC phenotype, paracrine activity, and exosome cargo. MSCs preconditioning with cytokines e.g. IFN-γ or TNF-α is known to enhance both immunomodulatory and exosomal functions and lower inter-donor variability [26]. These dimensions of heterogeneity illustrate the need to clearly state origin of MSCs, donor features, and culture parameters when comparing or evaluating therapeutic efficacy. The recognition and management of these diversities are essential to standardization, moving MSCs and MSC-Exos therapies closer to true precision regenerative medicine.

## 3 Strategies for enhancing MSCs and MSCs-Exosome function therapeutic efficacy: genetic modification, biochemical, and biomaterial Strategies

As previously mentioned, MSCs and their MSCs-Exos hold remarkable therapeutic potential across a wide range of diseases. However, their clinical efficacy is often limited by insufficient homing to injury sites, low survival after transplantation, and suboptimal differentiation *in vivo* [11]. To expand their therapeutic potential, using specific strategies that enhance survival, engraftment, homing, and paracrine activity *in vivo* are necessary. Following these main strategies including Genetic modification, non-Genetic modification, and Biomaterial application will be discussed.

### 3.1 Genetic modification of MSCs

Genetic modification represents a powerful strategy to overcome important naïve limitations and enhance the functional capacity of MSCs and MSCs-Exos. Through viral and non-viral gene transfer techniques, MSCs can be engineered to overexpress specific proteins, transcription factors, chemokines, or growth factors, thereby improving their migration, proliferation, and regenerative abilities [27,28]. Unlike unmodified or naïve MSCs with relatively general paracrine signaling, genetically modified MSCs can produce targeted therapeutic molecules, enabling more precise modulation of disease mechanisms [29]. Techniques include transient non-integrative approaches, such as plasmid DNA or RNA delivery, as well as genome-integrative strategies using viral vectors, including adenoviral (AVVs), lentiviral/retroviral (LVVs), and adeno-associated vectors (AAVs) for stable long-term expression [30]. Furthermore, CRISPR/Cas9 technology allows precise activation or silencing of genes in MSCs. For instance, HIF-1α upregulation via CRISPR enhances MSCs survival and their potency in hypoxic environments, while targeted deletion of factors like PDGF can improve differentiation and regenerative outcomes [31,32]. Genetic modification strategies aim to maximize therapeutic efficacy with minimizing cell alteration and death, offering a customizable approach to optimize MSCs function for regenerative medicine applications.

### 3.2 Non-genetic modification of MSCs

Non-genetic modification methods have been found to be clinically superior techniques for the augmentation of the therapeutic efficacy of MSCs and their exosomes. They avoid permanent alterations in the genome while indisputably augmenting function. Such methods mainly include pharmacological and small-molecule priming, stimulation with cytokines, and hypoxic preconditioning.

#### 3.2.1 Pharmacological and small-molecule priming

Pretreatment of MSCs with small molecules can enhance specific abilities, including immunomodulation, migration, and survival. For instance, valproic acid and lithium/S1P synergistically enhanced MSCs migration and neuroprotection [33], while ATRA pretreatment reduced pro-inflammatory cytokines (TNF-α/IL-17) and upregulated regenerative factors (VEGF/CXCR4), leading to improved outcomes in wound healing and emphysema models [34].

#### 3.2.2 Cytokine priming

Exposure to cytokines radically changes the therapeutic landscape of MSCs. TNF-α pre-exposure polarizes MSCs to an anti-inflammatory phenotype and expands immunoregulatory exosome secretion. [35]. Similarly, IL-1β potentiates the regenerative effect of MSCs-exosomes by inducing NRF2 activation in astrocytes, while IL-3 increases CXCR4 expression, augmenting chemotaxis towards SDF-1α gradients [36].

#### 3.2.3 Hypoxic preconditioning

Hypoxia mimics the physiological niche of MSCs (1–7% O_2_), unlike normoxic *in vitro* conditions. Hypoxic preconditioning boosts proliferation, glycolysis, genetic stability, and survival, while upregulating HIF-1α, CXCR4/7, and LincRNA-p21, thereby improving angiogenesis, migration, and exosome secretion [37,38]. Hypoxia-primed MSCs-Exos have shown superior efficacy in tissue repair, neuroprotection, and antifibrotic responses compared to normoxic controls [39]. MSCs-Exos, especially when MSCs are preconditioned (e.g., under hypoxia), show enhanced angiogenic effects *in vitro* and *in vivo* by upregulating growth factors such as Vascular Endothelial Growth Factor (VEGF), Hepatocyte Growth Factor (HGF), Fibroblast Growth Factor (FGF), and activating signaling pathways such as Protein Kinase B/Endothelial Nitric Oxide Synthase (AKT/eNOS) and Mitogen-Activated Protein Kinase (MAPK) [40]. These optimized exosomes also suppress pro-inflammatory cytokines (e.g., IL-1β, TNF-α) and promote anti-inflammatory cytokines (e.g., IL-10, TGF-β), shifting immune cell phenotypes (e.g., macrophages) towards pro-healing profiles, while reducing cell death through activation of survival pathways [1,41,42].

### 3.3 Biomaterials for MSCs and MSCs-Exos optimization

Recent advances have demonstrated that biomaterials represent a powerful and innovative strategy for optimizing MSCs and their derived exosomes for regenerative medicine. Unlike genetic or non-genetic modification approaches, biomaterials can improve MSCs survival, engraftment, paracrine secretion, and exosome biogenesis, while simultaneously serving as carriers to achieve controlled release and targeted delivery of EVs to diseased tissues [17,43]. This novel strategy introduces new opportunities in regenerative medicine, not only as supportive scaffolds but also as active modulators for optimizing MSCs and exosome-based therapies. For instance, hydrogels, scaffolds, and nanoengineered matrices have been shown to significantly increase exosome yield, preserve bioactivity, and promote tissue regeneration by fine-tuning immune responses and angiogenesis [44]. In this review, to establish a clear understanding, the following section will first provide an overview of **biomaterial classes and their properties.** Subsequently**,** we will discuss their role in **enhancing MSCs and exosome-based therapies** in detail to highlight their crucial role as next-generation regenerative strategies.

## 4 Classification of biomaterials: foundations for advanced regenerative applications

Biomaterials are engineered substances designed to interact with biological systems for therapeutic or diagnostic purposes, including tissue engineering, stem cell therapies, and drug delivery. Their classification based on origin, physicochemical properties, and functionalities is critical for selecting the most suitable materials for specific clinical applications. In the following sections, we provide a systematic classification of biomaterials, highlighting their distinct categories and relevance in regenerative medicine and cell-based therapies.

### 4.1 Metallic biomaterials

Metallic biomaterials are predominantly inorganic, have high mechanical strength, durability, and corrosion resistance, and can be either bioinert or bioactive depending on surface treatments. Their superior load-bearing capacity and fatigue resistance make them indispensable in orthopedic, dental, and cardiovascular implants. Advanced surface modifications as key factors for implant longevity and function include ion doping, hydroxyapatite coatings, and nanostructured composites. These modifications substantially improve osteogenic potential and antibacterial properties, notably enhance biocompatibility, and promote better bone integration and reduce implant-related infections. Applications include orthopedic artificial limbs, dental implants, cardiovascular stents, and surgical instruments. Titanium (Ti) implants, known for their excellent strength-to-weight ratio, corrosion resistance, and biocompatibility, are widely used in hip and knee replacements. Recent advancements in surface modification techniques have meaningfully enhanced the osteogenic and antibacterial properties of Ti implants. [45,46]. Metallic biomaterials play a pivotal role in optimizing MSCs and exosome-based therapies through their ion release, mechanical properties, and surface bioactivity. titanium-based scaffolds functionalized with MSCs-Exos have demonstrated improved bone regeneration and enhanced intercellular signaling, highlighting their translational potential in orthopedic applications [47]. Another research by Lu et al. reported that Coating metallic surfaces such as titanium with extracellular vesicles can increase MSCs adhesion, calcium-phosphate deposition, and osteogenic activity [48].

### 4.2 Ceramic biomaterials

Ceramics such as hydroxyapatite and bioactive glasses are widely utilized in regenerative biomaterials owing to their exceptional biocompatibility and osteo conductivity. These inorganic materials maintain chemical stability and low toxicity, and they can form direct bonding with bone tissue; however, their inherent brittleness and limited tensile strength restrict their use in load-bearing applications. In practical settings, ceramics are commonly applied in bone grafts, dental rest orals, and as bioactive coatings on implants. For example, hydroxyapatite coatings on orthopedic and dental implants encourage bone bonding and integration, while bioactive glass formulations stimulate bone regeneration via ionic dissolution and surface reactivity mechanisms. Recent advances highlight the evolving role of bioactive ceramics in medical devices and at tissue interfaces [49,50]. Wa et al. demonstrated that mesoporous bioactive glasses can enhance MSCs-Exos secretion, thereby promoting bone regeneration and immunomodulation in both *in vitro* and *in vivo* [51].

### 4.3 Polymeric biomaterials (Natural and synthetic)

Natural polymers and hydrogels provide excellent biocompatibility, biodegradability, and support cell adhesion, making them excellent scaffolds and delivery systems in regenerative medicine. Synthetic polymers allow precise control over mechanical and degradation properties, useful for diverse biomedical applications. For example, PCL is an FDA-approved flexible polyester with slow degradation, ideal for long-term scaffolds, while PLGA is commonly used for controlled drug release. Collagen-based hydrogels mimic the extracellular matrix to aid tissue repair. Hydrogels are three-dimensional polymer networks that absorb water, classified into natural, synthetic, and hybrid types [52]. Natural hydrogels come from biopolymers like chitosan and alginate, prized for bioactivity, while synthetic hydrogels, made from polymers like PEG and PVA, offer tunable mechanical properties. Hybrid hydrogels combine both advantages, enhancing biomedical functionality [53]. Zeng et al. developed recombinant human collagen methacryloyl (RHCMA) hydrogels to encapsulate exosomes from different MSCs sources, creating a bioactive matrix that enhanced wound healing. The hydrogel network improved cell proliferation, migration, and angiogenesis *in vitro* and significantly accelerated tissue repair *in vivo*. Importantly, RHCMA provided controlled release and stabilization of MSCs-Exos, thereby optimizing their therapeutic efficacy [54]. Shahzad et al. developed a novel immunotherapy through encapsulation of MSCs-Exos and a PPAR-γ agonist into PLGA microspheres for targeted treatment of allergic airway diseases. The artificial carrier offered sustained retention, slow release, and improved delivery to the inflamed respiratory tissue. Above all, PLGA encapsulation optimized the stability and functional activity of MSCs-Exos, enhancing their immunomodulatory potential [55]**.**

### 4.4 Composite biomaterials

Composite biomaterials integrate distinct classes of materials, including polymers and ceramics, to synergistically improve mechanical robustness and biological performance. By balancing structural durability, bioactivity, and controlled degradation, these composites address the multifaceted demands of tissue engineering applications. In bone regeneration, dental restorations, and orthopedic implants, combinations like polycaprolactone (PCL) with hydroxyapatite have demonstrated greater bone healing and mechanical reinforcement compared to monolithic constructs [56]. Advances in fabrication techniques, including additive manufacturing and microarchitectural design, allow precise three-dimensional control of porosity and phase distribution. The synergy of biodegradable polymers and bioactive ceramics fosters cellular adhesion, proliferation, and matrix deposition, rendering these composites critical in scaffold design for durable and functional tissue restoration [57]. Si et al. developed a composite hydrogel (MSCs-Exos/ZIF-8@GelMA) that included MSCs-Exos and metal-organic frameworks (ZIF-8) within a GelMA matrix to enhance bone repair. The hydrogel was highly biocompatible and supported hBM-MSCs adhesion, proliferation, and osteogenic differentiation. Interestingly, the composite biomaterial enabled stabilized and optimized MSCs-Exos delivery, which significantly accelerated bone regeneration *in vitro* and *in vivo* [58].

### 4.5 Smart biomaterials

Smart biomaterials are engineered to detect and respond to environmental stimuli such as pH, temperature, light, or electrical signals. This dynamic responsiveness enables precise therapeutic actions, controlled release of bioactive molecules, and active promotion of tissue regeneration. By adapting to local biological cues, these materials support personalized medicine and targeted interventions, minimizing systemic side effects. Examples include pH-sensitive hydrogels for selective drug release in acidic wound or tumor environments, and thermo-responsive polymers for minimally invasive, on-demand delivery. Integration with MSCs or MSCs-Exos further enhances regenerative outcomes. Advances in polymer design, hydrogel crosslinking, and stimuli-responsiveness highlight their transformative potential in modern regenerative therapies [59,60].

### 4.6 Nanoparticles (NPs)

In addition to the conventional five categories of biomaterials, nanoparticles (NPs) are recognized as a separate field within biomaterials, owing to their distinct properties and critical contributions to modern biomedical engineering, therapeutic delivery, and tissue interactions. NP (1–100 nm) exhibit unique properties due to their high surface area, reactivity, and nanoscale effects. They are classified as metallic (e.g., silver, gold), ceramic (e.g., nano-hydroxyapatite), and polymeric (e.g., PLGA), each offering distinct biomedical advantages. NPs enable targeted therapy and controlled drug release, enhancing treatment precision. Silver NPs provide antibacterial activity in wound dressings, while polymeric NPs sustain therapeutic delivery. Magnetic NPs allow remote, non-invasive interventions. Integration into composite biomaterials improves scaffold mechanics and bioactivity. Their nanoscale interactions with tissues support regeneration and tissue engineering. Continuous research explores novel NP formulations for advanced personalized medicine [61,62]. Mehta et al. explored the therapeutic potential of superparamagnetic iron oxide nanoparticles (SPIONs) curcumin-loaded in MSCs-Exos for the treatment of glioma. The strategy aimed to enhance the targeting efficiency and therapeutic efficacy of exosomes for treating glioma. The study indicated that SPION-curcumin-loaded exosomes could efficiently cross the blood-brain barrier and deliver drug agents to glioma cells, offering a potential therapeutic approach to glioma [63].

Following the general classification of biomaterials, their functional applications to enhance MSCs and exosome therapies are crucial. Biomaterials provide protective microenvironments that improve MSCs survival, regulate cell behavior, and stabilize therapeutic exosomes, ultimately enhancing regenerative efficacy. Xue et al. emphasized that biomaterials in stem cell therapy should be defined by their functional applications rather than composition. They categorized biomaterials into three key roles: (I) tracking transplanted cells, (II) modulating stem cell behavior, and (III) protective delivery to pathological tissues. This framework positions biomaterials as active facilitators of MSCs survival, differentiation, and paracrine function [64]. Similar studies demonstrate that advanced biomaterial platforms, including hydrogels, electrospun scaffolds, and nanoparticle systems, show the ability to maintain the functionality of MSCs and optimize exosome-mediated tissue repair [65]. These systems enhance **stability, controlled release, and microenvironmental signaling**, significantly boosting MSCs and MSCs-Exos therapeutic outcomes. We will then explore fabrication strategies, including electrospinning, hydrogel encapsulation, and nanoparticle-based delivery, that translate these advantages into practical applications of MSCs and MSCs-Exos.

## 5 Advanced fabrication techniques for biomaterial based MSCs and MSCs-Exos optimization

The efficacy of MSCs and MSCs-Exos based therapies is significantly influenced by the biomaterial scaffolds utilized, which administrate cell viability, paracrine function, and therapeutic delivery. Advanced fabrication techniques, including electrospinning, 3D bioprinting, and hydrogel encapsulation, provide precise control over scaffold architecture, porosity, and mechanical properties, establishing a microenvironment that supports stem cell survival and regulates exosome release. By integrating these advanced material strategies with stem cell biology, regenerative outcomes and translational potential are maximized [66].

### 5.1 Electrospinning

Electrospinning is a generally used cutting-edge technique for fabricating nanofibrous scaffolds that mimic the natural extracellular matrix (ECM), providing a conducive environment for MSCs attachment and proliferation. This feature makes electrospun fibers ideal for cell adhesion, proliferation, and differentiation, which are essential for tissue regeneration and healing. Moreover, this fiber network generates an optimal environment for biomedical applications, including wound dressings and stem cell therapy scaffolds [67,68]. Recent advancements have focused on optimizing electrospinning parameters to enhance the stability and functionality of encapsulated exosomes. For instance, incorporating electrospun nanofibers into hydrogel matrices has been shown to improve the mechanical properties and stability of the scaffolds, facilitating sustained release of exosomes and promoting tissue regeneration. Studies have demonstrated that electrospun nanofibers can effectively deliver stem cell-derived exosomes to target tissues, enhancing therapeutic outcomes in wound healing and tissue repair [69].

### 5.2 Hydrogel encapsulation

Hydrogel encapsulation of MSCs has emerged as a pioneering strategy in regenerative medicine, providing a hydrated, biocompatible microenvironment that preserves cell viability, enhances paracrine activity, and shields transplanted MSCs from the immune system and hostile conditions of the host [70]. Encapsulation of MSCs-Exos within hydrogels enables a controlled delivery and sustained release of bioactive vesicles, amplifying their therapeutic effects over time. The potential of hydrogel-exosome systems in tissue engineering applications, demonstrating their ability to promote tissue regeneration and chronic wound healing [71]. For example, recent studies have demonstrated that hydrogel-based MSCs and MSCs-Exos delivery systems, as a major innovation in tissue engineering and cell therapy, significantly enhance angiogenesis, neurogenesis, immunomodulation, and overall tissue repair in diverse pathological contexts [72]. On the other hand, stimuli-responsive hydrogels enable reacting to environmental triggers such as pH or temperature, thereby enhancing the temporal and spatial precision of therapeutic delivery. Furthermore, the incorporation of stimuli-responsive hydrogels allows for dynamic control over exosome release, enabling targeted and timely delivery of therapeutic agents. This approach enhances the precision and efficacy of MSC and MSCs-Exos based therapies, addressing challenges such as rapid clearance and off-target effects [73].

### 5.3 Three-dimensional (3D) bioprinting

3D bioprinting enables the precise fabrication of complex, patient-specific scaffolds with controlled architecture and mechanical properties. This technique allows for three-dimensional arrangement of cells and biomaterials, facilitating the creation of tissue constructs that closely mimic native tissues and the arrangement of MSCs and MSCs-Exos. Recent studies have explored the use of 3D bioprinted scaffolds for MSCs and exosome delivery, demonstrating their potential in promoting tissue regeneration and sustained release of exosomes, enhancing their therapeutic efficacy in wound healing applications [74].

Altogether, advanced fabrication techniques play a crucial role for preparing optimized MSCs and MSCs-Exos based therapies by providing controlled microenvironments and enabling precise delivery of therapeutic agents. These techniques improve the regenerative potential of MSCs and MSCs-Exos as promising strategies for tissue repair and regeneration.

## 6 Biomaterial-optimized MSCs and MSCs-Exos for multi-tissue regeneration

The integration of biomaterials with MSCs and MSCs-Exos can overcome the inherent limitations of cell-based therapy, including poor engraftment, rapid clearance, and uncontrolled paracrine signaling. Recent evidence highlights that biomaterial-optimized MSCs therapies, compared to MSCs alone, significantly improve regenerative outcomes in various regenerative areas, which are discussed below.

### 6.1 Pulmonary fibrosis

In preclinical models of lung injury and fibrosis, MSCs and MSCs-Exos embedded or delivered with biomaterial carriers, e.g., ECM-mimetic gels and exosome-enriched decellularized matrices, show superior local preservation and sustained bioactivity versus free injections, leading to reduced inflammation, lower myofibroblast activation, and decreased collagen fiber deposition. These optimizations directly address the major translational obstacle of rapid systemic clearance and poor localization in the lung [75].

### 6.2 Cardiac repair in myocardial infarction

MSCs or MSCs-Exos loaded hydrogels and injectable ExoGels function as an implantable/injectable agent that preserves vesicle integrity. This mechanism enables controlled release of pro-angiogenic and anti-fibrotic cargo, induces neovascularization, and improves functional endpoints when EVs are delivered from engineered hydrogels [76]. Xu et al. created an injectable hydrogel with MSCs-Exos to promote cardiac repair in a myocardial infarction model. The hydrogel enhanced angiogenesis, reduced cardiomyocyte apoptosis, preserved Connexin-43 expression, and improved ventricular remodeling and overall cardiac function [77]. Uman et al. showed that using *in vivo* tracking, EVs embedded in degradable hydrogels persisted in myocardium for more than 14 days, whereas EVs delivered in saline were cleared within 24h, underscoring the importance of carrier design for sustained bioavailability [78].

### 6.3 Neurological disorders

In CNS injury models**,** hydrogel encapsulation or adhesive hydrogel depots loaded with MSCs- Exos or neural stem cell-derived exosomes provide a local, sustained release that enhances angiogenesis, neurogenesis**,** and immune modulation across the blood–brain/spinal-cord barriers, producing measurable improvements in functional recovery versus systemic EVs dosing. Several experimental reports (ischemic stroke, TBI) directly demonstrate that EV-loaded hydrogels outperform free EVs delivery [79].

### 6.4 Musculoskeletal regeneration

By providing sustained local release, maintaining vesicle bioactivity, and promoting osteo/chondrogenic signaling, biomaterial-optimized MSCs-Exos, especially via hydrogels, improve bone and cartilage repair. For instance, a recent study demonstrated that bioglass/hydrogel scaffolds loaded with MSCs-Exos enhanced vascularized bone regeneration through effective miRNA delivery [80]. Other reports confirm that hydrogel encapsulation prolongs exosome preservation and functional efficacy in musculoskeletal tissue repair [81].

### 6.5 Wound healing and smart dressings

Integration of MSCs and MSCs-Exos with biomaterial-based wound dressings, which consist of hydrogels, electrospun nanofibers, and bioactive membranes, shows significant efficacy in enhancing cutaneous regeneration. These engineered combinations provide a proper niche to preserve MSCs viability, improve homing, and allow for controlled release of exosomes and growth factors. These results induce enhanced angiogenesis, collagen remodeling, and epithelialization, and reduced inflammation and scar formation [54].

Based on preclinical evidence in pulmonary, cardiac, neurological, and musculoskeletal systems, it is obvious that biomaterial-optimized MSCs and MSCs-Exos introduce a complex design for regenerative medicine. Moreover, wound dressings represent a highly promising field offering a modulable local microenvironment for long-term vesicle and cell functionality. In the following, the current review turns to focus on using biomaterial-optimized MSCs and MSCs-Exos in wound healing. The remaining parts concentrate on wound types, wound dressing architecture, and the latest research studies about biomaterial-optimized MSCs and MSCs-Exos for cutaneous wound regeneration.

## 7 Wound Classification and advanced wound dressing Strategies

### 7.1 Definition, classification, and phases of wound healing

The skin comprises three systematically organized layers: the epidermis, dermis, and hypodermis (subcutaneous tissue), each performing distinct tasks within the body. Skin damage by injury or dysfunction of skin tissue is commonly termed a wound [82]. Physical or chemical damage to the skin can cause wound formation, which is accompanied by discomfort, impairment, and hemorrhage. Wounds can be categorized as open, characterized by a breach in the skin that exposes internal tissue, or closed, where the skin remains intact, preventing exposure of the underlying tissues [83].

**Fig. 2:**
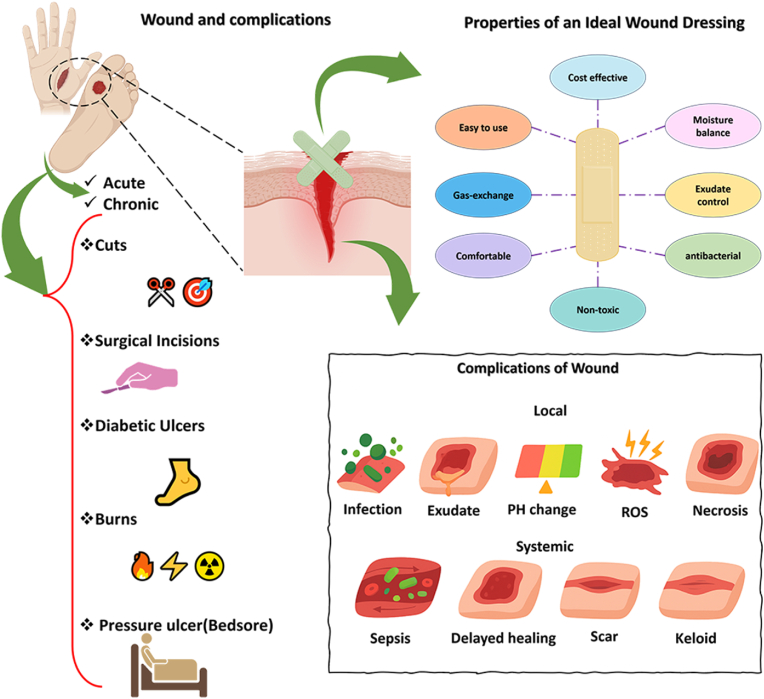
**Normal and chronic wound healing process.** Wound healing management is critical and related to the proper role of cytokines and inflammatory cells. The figures are original and were created using Microsoft Office PowerPoint 2021.

Wounds may also be categorized based on their genesis, length, level of contamination, depth, extent, location, and specific morphological characteristics [83]. The duration of healing will also be influenced by these parameters, permitting its categorization into acute, sub-acute, and chronic stages. Acute wounds can present a range of manifestations, from superficial abrasions to deeper lacerations. It is crucial to ensure that their development does not include the production of significant crust extension and that the healing period does not exceed 3 weeks [84,85]. Subacute wounds persist for a duration of three weeks to three months. Chronic wounds are typically infectious and frequently arise in immune compromised individuals. These wounds are susceptible to causes inducing tissue hypoxia and oxidative stress, as observed in diabetes, cardiovascular disease, hypothyroidism, hyperadrenocorticism, and malnutrition characterized by hypoproteinemia or deficiencies in vitamins and minerals. Severe and profound wounds situated in regions of significant tension may also become chronic. Wounds exposed to radiation and hypothermic conditions may potentially develop into persistent or chronic conditions. Infection constitutes the primary risk factor for the host's immune system [85,86]. Fig. 2 depicts a comparison between the wound healing process in normal and chronic skin wounds. Due to unusual remaining in the inflammation phase, the initiation of the chronic wound process has started with inflammatory cells and cytokines.

The Wound Healing Society divides chronic wounds into four types based on their cause: pressure ulcers, venous ulcers, arterial insufficiency ulcers, and diabetic ulcers. Chronic wound healing causes and symptoms are more complex and severe than those associated with regular wound healing [87].

The protracted maintenance necessary for chronic wounds augmented the financial strain on healthcare systems. A retrospective examination of medicare 5 % data set from 2014 indicates chronic nonhealing wounds affect approximately 8.2 million (15 %) medicare enrollees [88,89]. Medicare cost forecasts for all wounds varied between $28.1 billion $96.8 billion, with outpatient costs ($9.9–$35.8 billion) exceeding inpatient costs ($5.0–$24.3 billion) [90]. Furthermore, the 5-year death rate for diabetic foot ulcers (30.5 %) is comparable to that of cancer (31 %).

### 7.2 Conventional and advanced wound dressing approaches

Wound healing is a dynamic and interactive process comprising four phases: coagulation and hemostasis, inflammation, proliferation, and remodeling with scar tissue formation. The four steps facilitating wound closure entail interactions among various cell populations, soluble mediators, cytokines, and others, with the healing process commencing shortly following the injury. Chronic inflammation in the wound microenvironment, resulting from oxidative stress, decreased angiogenesis, heightened release of proinflammatory cytokines, and bacterial infection, may lead to fibroblast senescence [84,91]. Injured skin must be covered by an appropriate wound dressing to mitigate damage and infection risk, lower available oxygen, decrease pH, and expedite the restoration of tissue integrity. In recent decades, numerous biomaterials have been developed and marketed as skin substitutes [92].

**Fig. 3:**
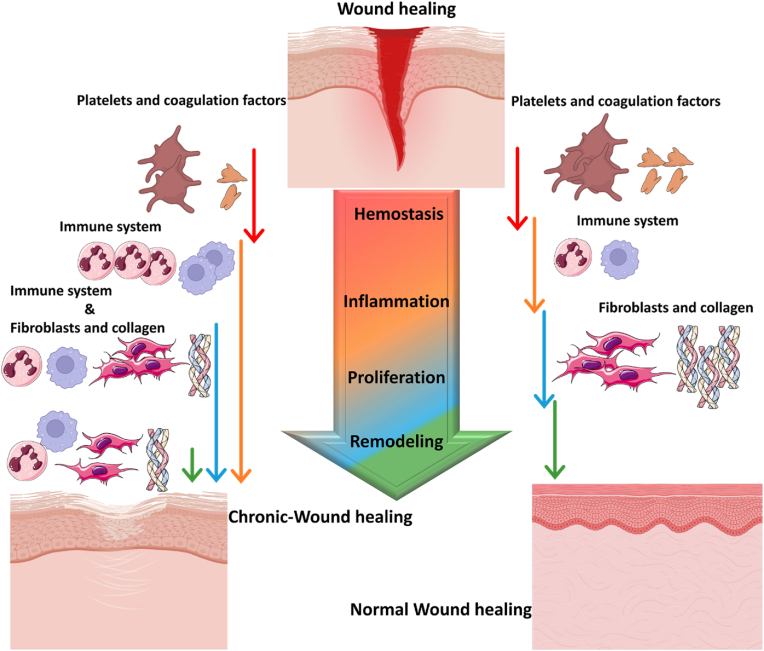
Comprehensive overview on wound classification, formation, complications, and characteristics of proper wound dressing to diminish the disadvantages of wounds. The figures are original and were created using Microsoft Office PowerPoint 2021.

The selection of optimal wound dressing depends on the kind of wound, depth, location, extent, degree of exudation, presence of infection, and the adhesion materials of the wound dressing. Also, an optimal dressing should combine affordability, non-antigenicity, durability, flexibility, non-toxicity, and non-adherence, while being easy to apply and remove, suitable for single-use procedures, non-traumatic, and composed of minimally processed biological materials [83]. On the other hand, it should also prevent water loss, adapt to irregular wound surfaces, facilitate gas exchange and exudate removal, provide mechanical protection, prevent contamination, and be biodegradable. Collectively, these attributes lead to faster, safer, and more effective wound healing, while maintaining patient comfort and minimizing the risk of complications [92,93]. Dressings can be categorized into three main classifications based on their material composition and physical forms: biological (of animal or plant origin), biological synthetic, or purely synthetic [94]. Coatings can be classified based on their physical form as oils, films, foams, hydrogels, hydrocolloids, or membranes [95]. By maintaining a moist environment, efficiently managing exudates, and protecting against infection, these wound dressings, when combined with cell therapy, can help to accelerate healing, reduce complications, and promote optimal tissue repair [96]. Here, the design and development of biomaterials along with cell therapy as a modern generation of wound dressing to accelerate wound contraction are critically required. Fig. 3 illustrates the comprehensive overview of wound formation, complications, and characteristics of proper wound dressing.

Integrating MSCs as a cell therapy and MSCs-Exos into wound dressings improves wound healing by boosting tissue regeneration, lowering inflammation, and re-epithelialization MSCs release exosomes containing bioactive chemicals that promote intercellular communication, angiogenesis, and extracellular matrix remodeling, and ultimately boost healing results [97]. AD-MSCs and UC-MSCs exosomes combined with hydrogel systems demonstrate some of the most remarkable wound healing accelerations, while bone marrow MSCs-seeded scaffolds consistently show the highest standardized wound healing improvements across multiple studies [98]. Furthermore, combining these therapeutic agents with advanced biomaterials such as hydrogels and scaffolds results in a bioengineered wound dressing that allows for controlled and sustained release of exosomes at the wound site, maximizing their therapeutic potential and providing a modern and particularly effective approach to regenerative medicine [99]. Advantages of using biomaterial-optimized MSCs and delivered MSCs-Exos are listed below through multiple mechanisms:

• Enhanced survival of MSCs and MSCs-Exos by protective scaffolds to mitigate apoptosis and oxidative damage.
• Improved retention and engraftment of MSCs and MSCs-Exos physically by hydrogel matrices at the wound site.
• The immunomodulation effect of MSCs secreted cytokines and MSCs-Exos reduces extreme inflammation and promotes tissue regeneration.
• Controlled release of growth factors, such as VEGF, TGF-β, FGF, and bioactive exosomal cargo accelerates angiogenesis, collagen deposition, and epithelialization.

This targeted, multifunctional approach results in faster wound closure, improved tissue architecture, and reduced scar formation compared to MSCs or MSCs-Exos delivered without carriers. In the following section, we highlight key studies focused on the above multiple mechanisms that integrate MSCs or MSCs-Exos within biomaterial platforms for optimization, such as hydrogels and scaffolds, showcasing their enhanced regenerative effects and superior wound healing outcomes in preclinical models.

## 8 Biomaterial-optimized MSCs and MSCs-Exos therapies: preclinical evidence for enhanced wound healing

MSCs or MSCs-Exos optimized by scaffold or hydrogel biomaterial as a unique wound dressing show maintaining viability, paracrine activity, and targeted delivery, which collectively dictate wound healing outcomes. These multifunctional dressings can also coordinate cellular responses, which promote tissue remodeling and regeneration. For chronic, complicated healing wounds, powerful adaptability and bioactivity underscore their translational potential, establishing bilayer dressings as a cutting edge approach to regenerative wound care [100]. Table 1 summarizes studies about MSCs embedded in hydrogel system therapies for wound healing and other tissue repair and regeneration.

**Table 1:** Hydrogel-based stem cell therapies for tissue repair and regeneration.

Hydrogel	Cell type	Key Features/Functions	Model	Reference
PEG–PLGA–PEG (thermosensitive)	MD-MSCs	Improves wound healing and supports regenerative cell growth	Diabetic db/db mice	[101]
Hydroxyapatite/Chitosan	BM-MSCs	Activates angiogenesis and accelerates wound closure	Sprague rats	[102]
Hyaluronidase-modified spongy hydrogel	AD-MSCs	Encourages nerve regeneration; lowers macrophage infiltration	Mice	[103]
Gel–PEG	AD-MSCs	Reduces inflammation; stimulates neovascularization	Diabetic db/db mice	[104]
Hydrogel sheet	AD-MSCs	Faster wound closure compared to controls	Diabetic patients	[105]
Chitosan–polyurethane	BM-MSCs	Promotes bone repair and reduces inflammation	Rats	[106]
N-chitosan + Hyaluronic acid	BM-MSCs	Stimulates secretion of anti-inflammatory factors	SD rats	[107]
Porcine submucosal hydrogel	BM-MSCs	Enhances proliferation, migration, and angiogenesis	Rats	[108]
Gelatin–sericin	AD-MSCs	Protects against ROS damage; promotes angiogenesis	Wistar rats	[109]
Antioxidant polyurethane	AD-MSCs	Prevents infection; enhances fibroblast proliferation and granulation	Rats	[110]
Pluronic F-127	UC-MSCs	Upregulates VEGF and TGF-β1	Rats	[111]
Collagen–PEG	UC-MSCs	Reduces TNF-α; increases VEGF expression	Rats	[112]
Pluronic F-127	WJ-MSCs	Enhances dermal regeneration and collagen deposition	Rats	[113]

Recent studies have highlighted the combination of MSCs and MSCs-Exos with biomaterials that demonstrated significant gains in wound healing. Aghayan et al., by a comprehensive meta-analysis of 23 preclinical studies, showed that BM-MSCs combined with scaffolds resulted in the largest wound closure effects, especially in early stages of healing at one-week post-treatment. Following this synergistic usage, improvement of wound closure rates is accompanied by enhanced collagen deposition and angiogenesis. The scaffolds provide mechanical support, improve MSCs engraftment, and modulate inflammation, thereby accelerating tissue repair [114]. Recent preclinical studies show that incorporating MSCs or MSCs-Exos into smart biomaterials such as hydrogels and porous scaffolds can improve cell retention, prolong therapeutic effects, and underscore that this multifunctional combination has the potential to accelerate wound repair in different animal models.

Yang et al. engineered an injectable hyaluronic acid hydrogel loaded with MSCs-Exos as a bioactive wound dressing. This optimized hydrogel system enhanced exosome stability and preservation, stimulated angiogenesis, collagen fibers deposition, and significantly accelerated wound closure in full-thickness skin injury models [115]. Deng et al. demonstrated that hyaluronic acid-based hydrogels can act as smart wound dressings by optimizing exosome delivery from MSCs. These bioactive hydrogel dressings protect and gradually release MSCs-Exos at the wound site, maintaining their bioactivity and enhancing angiogenesis, fibroblast migration, and collagen remodeling. Finally, this dressing can lead to dramatically faster and more effective healing of skin wounds [116] AD-MSCs-Exos encapsulated within alginate hydrogels also demonstrated strong regenerative activity *in vivo*. Shafei et al. demonstrated that this composite platform boosted full-thickness wound healing, enhanced angiogenesis, and promoted collagen production compared to controls. The hydrogel scaffold preserved exosomal efficacy and enabled local sustained delivery, circumventing limitations of short clearance and cell viability. By utilizing paracrine signaling in a cell-free setup, this technique reduced the risk of immune rejection while maintaining therapeutic efficacy. These findings demonstrate the promise of exosome–hydrogel platforms as the next generation of wound dressings [117].

An ideal biomaterial platform with improved stability and regenerative potential for tissue repair is exosome-hydrogel (Exo-gel), a hydrogel system that contains MSCs-Exos. Through spatiotemporally controlled release, enhanced extracellular matrix remodeling, angiogenesis, and nerve regeneration, MSCs loaded hydrogels or Exo-gel composite systems significantly improve regenerative therapy in preclinical wound dressing studies for ulcer [118].

Umbilical Cord-derived MSCs (UC-MSC) Exosomes embedded in pluronic F-127 hydrogels have shown substantial ability to accelerate wound healing in diabetic and chronic wound models. This hydrogel platform ensures sustained exosome delivery in the wound position, promoting re-epithelialization and granulation tissue formation through enhanced release of key growth factors such as VEGF and TGF-β [111]. Li et al. showed exosome-loaded genipin-crosslinked hydrogels enhance full-thickness cutaneous wound healing in rats. Long-term release of the hydrogel enhanced fibroblast proliferation, migration, and angiogenesis. Anti-inflammatory and pro-regenerative characteristics also facilitated optimal tissue repair. The work determines the therapeutic advantage of using exosomes with engineered hydrogels for effective wound regeneration [119].

In a recent study by Keshavarz et al., encapsulated Adipose-Derived MSCs (AD-MSCs) spheroids in gelatin hydrogels accelerated wound healing compared with controls after 14 days. This combination significantly improved angiogenesis and epidermal regeneration via enhanced secretion of angiogenic growth factors such as VEGF and PDGF. Histopathological analysis revealed increasing epidermal thickness compared to suspension-based treatments, emphasizing the beneficial effect of spheroid culture in hydrogel matrices [120]. Zhang et al. developed a sodium alginate/collagen hydrogel seeded with human umbilical cord MSCs (hUC-MSCs) for enhanced wound repair. The hydrogel accommodated an integrative scaffold that improved MSC survival and retention. Their system improved fibroblast proliferation, migration, angiogenesis, and extracellular matrix deposition. Anti-inflammatory activities with the modulation of cytokine signaling and NLRP3 inflammasome inhibition facilitated tissue repair. This work holds the promise of co-culture of hUC-MSCs with biomimetic hydrogels to achieve efficient skin regeneration [121]. Sprayable, thermosensitive exosome hydrogels improve coverage of irregular defects and sustain vesicle release, accelerating epithelial closure and granulation in burn wounds [122]. Microparticle platforms using glycyrrhizic-acid hydrogels to encapsulate MSCs exosomes add on-demand, near-infrared triggered release and combine antibacterial, antioxidant, and pro-angiogenic actions, effective even in infected diabetic wounds [123,124]. Recent studies indicate that MSCs-seeded hydrogels enhance healing in diabetic wounds, while exosome-loaded nanofiber scaffolds yield superior healing outcomes compared to control dressings. Together, these findings reveal the potential of combining biomaterials with both MSCs and MSCs-Exos as emerging wound therapeutics [125,126]. Smith et al. showed that MSCs generated from human adipose tissue placed in hydrogels greatly improve wound healing. The hydrogel maintained MSC viability and encouraged growth factor secretion for an extended period of time. This encouraged the migration, proliferation, and deposition of extracellular matrix by fibroblasts. Additionally, the anti-inflammatory benefits were enhanced by the biomaterial scaffold. All things considered, MSCs capacity to regenerate chronic wounds is enhanced when combined with hydrogels that have been optimized [127]. Also, results from some studies, e.g., Krasnodembskaya et al., demonstrated that human MSCs secrete LL-37 with direct anti-staphylococcal activity, which supports the antimicrobial arm of MSCs-based dressings [[128], [129], [130]].

## 9 Challenges and future perspectives in optimized MSCs and exosomes applications

Optimized MSCs and their derived exosomes exhibit remarkable regenerative potential through multiple mechanisms, including promotion of angiogenesis, modulation of immune responses, and protection against apoptosis [41]. Unlike MSCs, which require strict cryopreservation and lose potency during freeze-thaw cycles, exosomes can be preserved by ultra-low freezing, lyophilization, or incorporation into stabilizing excipients [131]. Good Manufacturing Practice (GMP) compliant protocols are being developed to ensure reproducibility in size, purity, and bioactive composition, with harmonized standards for potency assays, sterility, and marker profiling [132]. Despite their promising regenerative capacity, translating optimized MSCs and MSCs-Exos into reliable clinical therapies faces several practical and regulatory challenges.

### 9.1 Strategies to minimize NPs toxicity in MSCs and MSCs-Exos based therapeutics

NPs hold great promise in both regenerative medicine and cancer therapy, but concerns about toxicity often limit their clinical translation. The biological impact of NPs is strongly influenced by their physicochemical properties, including size, surface charge, and degradability, which can determine whether they induce oxidative stress, DNA damage, or unwanted immune responses. Therefore, biocompatible design and careful functional optimization are essential to balance therapeutic efficacy with safety [133]. One promising approach to minimize nanoparticle-induced toxicity is to modify their surface with biocompatible coatings such as polyethylene glycol (PEG), chitosan, or other polymers. For example, PEG-coated silver NPs demonstrate reduced oxidative stress and improved biocompatibility compared to uncoated forms [134]. Another strategy is to utilize biodegradable nanomaterials, including lipid-based or polymeric nanoparticles, which degrade safely in the body and reduce concerns about long-term accumulation. Careful optimization of size, dose, and route of administration further lowers risks of cytotoxicity and off-target effects [135]. Importantly, these toxicity considerations are directly relevant when nanoparticles are integrated with MSCs, either by using MSCs as living carriers of NPs or by embedding MSCs within NP-based scaffolds. Both strategies rely on fine-tuned NP design to maintain MSC function and minimize adverse effects, making the study of NPs toxicity central to advancing MSCs-NP systems. Table 2 summarizes important challenges and probable resolutions in the field of MSCs-NPs systems.

**Table 2:** Key challenges and potential solutions in MSCs-NPs systems.

Challenge	Description	Possible Solutions	Reference
Maintaining the MSCs function after NPs loading	High NPs/drug load can impair MSCs viability, proliferation, differentiation, and migration.	Use biodegradable/PEGylated NPs; optimize dose–response; employ mild loading methods (e.g., surface conjugation or stimuli-responsive carriers).	[134]
Premature drug release & off-target accumulation	NPs may release payload before MSCs reach the target, causing systemic toxicity.	Engineer-controlled/stimuli-responsive release systems (pH, redox, ultrasound, light); improve NPs coating stability.	[135]
NP-scaffold compatibility (for regenerative delivery)	Some scaffolds release toxic degradation products or alter MSCs phenotype.	Use biocompatible hydrogels and nanocomposites; optimize scaffold degradation kinetics; functionalize scaffolds with ECM-like molecules.	[[136], [137], [138]]
Regulatory complexity/Manufacturing & scalability	MSCs-NPs systems are classified as both ATMPs and nanomedicines, complicating approval. Challenges arise in scaling NPs production and MSCs loading while maintaining consistency.	Develop standardized GMP protocols, conduct early regulatory consultation, and harmonize classification criteria. Develop automated cell–NP loading systems; implement robust quality control; apply scalable NP synthesis under GMP	[139,140]
Long-term safety	Limited *in vivo* data on the chronic fate of NP-loaded MSCs or MSC-laden scaffolds (tumorigenicity, ectopic differentiation, NPs accumulation)	Conduct long-term preclinical tracking; use biodegradable NPs; apply rigorous safety assays (genotoxicity, biodistribution)	[141,142]
Heterogeneity in study designs	Differences in NP types, MSC sources, administration routes, and disease models hinder reproducibility	Establish consensus protocols for characterization (size, zeta potential, coating); standardize MSC source and functional assays	[[143], [144], [145]]

### 9.2 Enhancing MSCs stability and therapeutic efficacy

MSCs tend to lose potency during long-term culture; therefore, strategies to enhance their survival and functionality are essential. Genetic modification (e.g., overexpression of anti-apoptotic or angiogenic genes) can improve therapeutic potency, although safety must be carefully monitored. Preconditioning approaches, such as hypoxia or cytokine stimulation, boost the secretion of beneficial growth factors and immunomodulatory molecules. In addition, biomaterial encapsulation of MSCs within hydrogels or scaffolds protects them from hostile microenvironments and enables sustained release of therapeutic factors [146].

### 9.3 Exosome optimization and delivery challenges

The therapeutic effects of MSC-Exos are mediated by cargo components primarily miRNAs, proteins, and lipids that act synergistically on target cells. miRNAs such as miR-21, miR-146a, and miR-126 regulate angiogenesis, inflammation, and apoptosis by modulating key signaling pathways (e.g., PI3K/AKT, NF-κB, MAPK) [147,148]. Exosomal proteins, including growth factors (VEGF, HGF, FGF2) and tetraspanins (CD9, CD63), contribute to intercellular communication, immune modulation, and tissue regeneration. Evidence suggests a cooperative mechanism in which miRNAs reprogram gene expression while proteins and lipids provide structural and signaling cues that enhance repair. Understanding these multi-layered interactions is crucial for designing optimized, targeted exosome therapies [147,149].

Exosome therapy can be enhanced by engineering vesicles with targeted ligands, allowing more precise delivery to diseased tissues. Stability is another critical issue; methods such as lyophilization, cryopreservation, and incorporation into stabilizing carriers (e.g., hydrogels or nanoparticles) improve storage and transport. Advances in bioengineering also enable tailoring exosomal cargo (e.g., loading therapeutic RNAs or proteins) for specific clinical applications [150].

### 9.4 Regulatory and manufacturing standardization in MSC/exosome research

The lack of unified protocols for MSC and MSCs-Exos production remains a barrier to translation. Current efforts focus on developing guidelines for isolation, characterization, and potency testing, ensuring reproducibility across laboratories. Variability in donor source, culture conditions, passage number, and exosome isolation techniques significantly affects product consistency, highlighting the need for harmonized protocols. Regulatory agencies increasingly emphasize GMP requirements, including sterility, identity, purity, and potency assays, with potency testing seen as essential for linking product characteristics to therapeutic activity [151,152]. International collaborations between academia, industry, and regulatory bodies are underway to harmonize safety and efficacy standards, paving the way for clinical applications [27,132,152]. These efforts include scalable and reproducible GMP pipelines for exosome and MSC manufacturing, quality validation of final drug products, and development of standardized functional assays such as immunomodulatory capacity and angiogenic activity [153,154]. Harmonized regulatory frameworks, including classification of exosome therapeutics as advanced therapy medicinal products in Europe and biologics in the US, are being actively discussed to ensure the product meets required safety and efficacy endpoints [155,156]. Such harmonized regulatory and manufacturing efforts are expected to accelerate the safe and effective clinical adoption of MSCs and exosome-based therapies.

### 9.5 Future perspectives

MSCs and MSCs-Exos based therapy, biomaterials, and nano engineering working together could lead to smarter, more effective, and more controllable next-generation therapies. For example, engineering hybrid delivery systems that combine exosomes with nanoparticle systems (to improve targeting, controlled release, or imaging) makes it possible to target with precision and reduce off-target effects. Advanced scaffold materials and 3D bioprinting also provide platforms for embedding MSCs or their MSCs-Exos in engineered matrices, allowing for spatial control and responsive behavior, such as responding to local cues like pH or inflammation. These kinds of integrated platforms could be very useful for repairing and regenerating tissue. Recent reviews declare this trend to be an important path for MSCs-derived exosome therapies. [139,157,158]. Personalized MSC/exosome therapy represents another critical frontier. Using patient-derived MSCs or customizing exosomal cargo (RNAs, proteins, ligands) to the patient's disease profile can improve efficacy and reduce immune or adverse responses. selecting MSCs from sources that match the patient's age, health status, and possibly even genetic background can reduce heterogeneity and improve therapeutic consistency [159,160].

Smart wound dressings and engineered matrices incorporating MSCs or exosomes are expected to become more clinically viable. Embedding exosomes in hydrogels or nanofibrous scaffolds enables localized, sustained release of regenerative factors at wound sites, which is particularly useful in chronic wounds, burns, or diabetic ulcers where standard treatments are insufficient. Bioengineering advances allow the dressings to respond to environmental cues (moisture, infection) or degrade in a controlled fashion. Some studies already show accelerated re-epithelialization, reduced scarring, and improved collagen deposition when using MSCs-Exos in such dressings [159,160].

Scalability, reproducibility, and clinical translation remain critical challenges but are also areas where significant progress is being made. Moving from small-scale preclinical studies to GMP-grade production demands standardizing MSCs culture conditions (passage number, oxygen tension, donor source), exosome isolation and purification methods, and functional potency assays. Regulatory considerations (safety, consistency, storage, delivery routes) need to be resolved so that therapies can reliably move from lab to bedside. Ma et al. highlight these requirements and give perspectives on establishing manufacturing pipelines, quality control, and safety testing [139]. Another future outlook is improving the targeting specificity and biodistribution of MSCs-Exos. Engineering exosomal surface ligands, or combining with targeting moieties (antibodies, peptides), may help direct exosomes more precisely to diseased tissues or cell types (inflamed endothelium, tumor microenvironment). Also, strategies to slow exosome clearance (modifying exosome membranes, using sustained release systems, encapsulation) are under consideration to extend half-life *in vivo* and allow lower dosing. Some of these ideas are discussed in reviews of MSCs-Exos biodistribution and challenges around fast clearance [157,159,161].

Finally, long-term safety, ethical, and regulatory frameworks will shape whether MSC/exosome therapies truly reach widespread use. Longitudinal studies in relevant animal models and early human trials need to monitor not just efficacy but possible adverse effects (immune reactions, oncogenic risk, unintended tissue interactions). Ethical considerations include donor selection, consent, and potential off-target effects. Regulatory bodies will likely require stricter definitions of "manufacturing" vs "modification," robust potency assays, stability and storage standards, and validated routes of administration. Building these frameworks and ensuring collaboration among researchers, clinicians, industry, and regulators are critical issues. Several recent articles emphasize that while the promise is considerable, the path to routine clinical use is nontrivial [139,159,161].

## 10 Conclusion

MSCs and MSCs-Exos represent a rapidly developing field in regenerative medicine, offering unique advantages in modulating immune responses and enhancing tissue repair. However, their clinical usage has been limited by some critical challenges, including inadequate stability, poor maintenance at target sites, uncontrolled release, and the hostile nature of pathological microenvironments such as chronic wounds and fibrotic tissues. Nanoparticles, stimuli-responsive hydrogels, and multipurpose smart delivery systems make up the integrated biomaterial system, which has created new avenues for breaking through these obstacles. These cutting-edge techniques greatly improve the therapeutic effectiveness of MSCs and MSCs-Exos and promote the clinical use of regenerative therapies. The biosafety of nanomaterials, heterogeneity among exosome batches, large-scale production, and regulatory standardization are some of the issues that still need to be resolved despite recent advancements. Multidisciplinary cooperation between clinical regulation, MSCs biology, and biomaterials technology is needed to address these issues.

## Author contributions

Fatemeh Rafati: Writing - Original Draft, Zeynab Ghorbani: Writing - Original Draft, Tahereh Manoochehrabadi: Writing - Reviewing & Designing, Farbod Khosravi: Writing - Original Draft, Writing- Review & Editing, Jila Majidi: Writing - Original Draft, Fatemeh Eskandari: Writing - Original Draft, Writing, Mohamad Eftekhary: Writing - Original Draft, Writing, Hajar Nasiri: Conceptualization, Project administration, Supervision, Writing - Review & Editing.

## Ethical approval

Not applicable.

## Funding

Non-funding.

## Conflict of interest

The authors declare no conflict of interest.

## Data Availability

No datasets were generated or analysed during the current study.
